# Self-Efficacy Mechanism in Farm Tourism Microentrepreneurship

**DOI:** 10.3389/fpsyg.2022.875096

**Published:** 2022-04-26

**Authors:** Bruno Ferreira, Duarte B. Morais, Susan Jakes, Gene Brothers, Craig Brookins

**Affiliations:** ^1^School of Community Resources and Development, Arizona State University, Phoenix, AZ, United States; ^2^HU-ASU International Tourism College, HAITC – Hainan University, Haikou, China; ^3^Department of Parks, Recreation and Tourism Management, North Carolina State University, Raleigh, NC, United States; ^4^NC State Extension, North Carolina State University, Raleigh, NC, United States; ^5^Department of Psychology, North Carolina State University, Raleigh, NC, United States

**Keywords:** entrepreneurial self-efficacy, microentrepreneurship, social capital, agritourism, entrepreneurship, permatourism

## Abstract

Tourism microentrepreneurship is an important farm diversification strategy, also contributing to the competitiveness of the destination. However, psychological and structural constraints seem to hold back farmers when it comes to starting or expanding tourism operations. We argue that social capital derived from farmers’ social networks affords sources of entrepreneurial self-efficacy, which boosts entrepreneurial intention. Analysis of survey data from 207 farmers in North Carolina revealed an adequate SEM model fit and strong significant relationships between bridging social capital and tourism microentrepreneurial self-efficacy. Internal self-efficacy factors were strongly and significantly associated with entrepreneurial intention. Triangulation with qualitative data from participatory-action research reinforced the importance of informal networking processes to model entrepreneurial behavior that boosts self-efficacy and reaffirms microentrepreneurial intentions. However, external self-efficacy was not significantly associated with farmers’ entrepreneurial intentions, which may be attributable to ambiguous agritourism policy.

## Introduction

There is a growing call for empirically based and theoretically driven solutions to support tourism microentrepreneurship initiatives, which stand to enhance the authenticity, uniqueness, and competitiveness of the destination (Hallak et al., 2012; Morais et al., 2017a; Çakmak et al., 2019; Destinations International, 2019; KC et al., 2021). At the same time, signature farm tourism is emerging as one of the most promising niches for tourism microentrepreneurism (Ferreira et al., 2020) by capitalizing on the popularity of the foodie scene among the populace (Weiss, 2012; Morais et al., 2017b). Agritourism prominently contributes to the diversification of farm income (Barbieri, 2017), to the preservation of productive agricultural lands (Vafadari, 2013), and the empowerment of rural women (Garcia-Ramon et al., 1995; Halim et al., 2020; Savage et al., 2020). However, perceived personal and structural barriers hinder farmers’ chances of success (Barbieri and Streifeneder, 2019; Ohe, 2020). For example, tourism is still uncharted territory for many farmers whose expertise lies predominantly in agricultural production (Mikko Vesala et al., 2007; Haugen and Vik, 2008; Joyner et al., 2018). Also, destination management organizations (DMO) have not consistently integrated farm experiences into the mainstream tourism product (McGehee, 2007; Ferreira et al., 2020).

In the current work, we seek to explore the effect of the strength of farmers’ symbiotic relationships in the tourism business ecosystem on their agritourism business intentions. In detail, we posit that bridging social capital affords vital sources of entrepreneurial self-efficacy, which increases entrepreneurial intention. This study is unique in three different ways. Firstly, to our knowledge, the effect of farmers’ concerns with visitor safety and legal liability on intentions to engage in agritourism has not been empirically examined, although it is widely assumed it is a detractor by academicians and practitioners alike. Secondly, while previous research may have either over or under-socialized farmers’ entrepreneurial behavior, this study examines the integrated influence of social capital and personal outcome expectations on the formation of entrepreneurial intentions. Third, it is the first time the permatourism community and tourism integrated model have been tested empirically. In sum, by explicating the combined role of farmers’ weak ties and outcome expectations in various areas of their purview, this paper aims to contribute to farm tourism microentrepreneurship initiatives’ continued development and success.

## Background

### Farm Tourism

Over the last decades, there has been an increased demand and supply of farm tourism experiences (Barbieri et al., 2016; Ohe, 2020). On the demand side, agritourists seek educational and recreational experiences, quality of life enhancement and socialization opportunities, and relaxation (Srikatanyoo and Campiranon, 2010; Barbieri, 2017). A sense of nostalgia evoked by farmscapes also seems to drive some visitors who desire to reconnect with the past (Joyner et al., 2018). On the supply side, despite strong non-pecuniary motivations (Quella et al., 2021), many farmers look at agritourism as an indispensable income source to retain family farmland and lifestyle in the face of financial pressure (Ollenburg and Buckley, 2007). The continued price drop of commodity crops (Barbieri and Mahoney, 2009) as well as the demise of tobacco farming in some states (Benson, 2008) are well documented threats to the traditional production-oriented farm. Hence, many small-scale farmers pivoted to specialty crops, selling their products directly to discerning consumers on and off-farm at a premium (Schilling et al., 2012; Chase et al., 2018; McKee, 2018). High-end farm-to-table restaurants also considerably source from small farms, not only for freshness and flavor but also to appease an increasingly socially and environmentally conscious clientele (Morais et al., 2017b). In turn, these *foodies* have shown a desire to visit local farms to educate themselves about their local food system (i.e., where their food comes from; Morais et al., 2017b; Chase et al., 2018).

However, many farmers feel that tourism detracts from their traditional farming lineage. For example, Ohe (2018) found that Japanese farmers with a more traditional farming identity were reluctant to charge for experiences, providing these services merely because they perceived social responsibility. Similarly, in Finland, farmers with a diversified portfolio of income-generating farm-related businesses (i.e., portfolio farmers) had higher perceived entrepreneurial identities than traditional farmers but lower entrepreneurial identities than other non-farm entrepreneurs (Mikko Vesala et al., 2007). Additionally, many farmers are reluctant to open up their farms to visitors because they are concerned with liability (Centner, 2010; Pegas et al., 2013; Ferreira et al., 2020). This has prompted rural development agencies such as Cooperative Extension to train farmers on improving their operations and adopting best practices in farm safety and liability management (Infante-Casella et al., 2018).

Furthermore, farmers’ assumptions of appealing activities do not always align with tourists’ rural imaginary and desired experiences, leading to ineffective marketing campaigns and low visitor satisfaction (Joyner et al., 2018; Nazariadli et al., 2019). Finally, they worry that agritourism will drain time and resources from farming and are doubtful that there is any money in farm tourism (Schilling et al., 2014). In sum, the literature suggests that farmers perceive many entry barriers, and therefore, they are often only lukewarm about pursuing agritourism (Haugen and Vik, 2008; Galluzzo, 2018). Hence, there is a pressing need for effective strategies that support farmers’ intentions to diversify into agritourism, for there is evidence that the economic and non-economic benefits afforded by diversification are fundamental to the viability of farms.

### Social Capital

Research on community-based tourism has consistently identified the critical role of social capital in influencing individuals’ involvement in tourism entrepreneurship (Jones, 2005; Pawson et al., 2017; Diedrich et al., 2019; Musavengane, 2019). Social capital is a dominant theory in the broader social sciences, widely accepted as a successful theoretical perspective to understand social relations (Narayan and Cassidy, 2001). The concept also found its way into everyday language through general circulation magazines, rapidly becoming a panacea to all ills afflicting society (Portes, 1998). It has been widely used in tourism research, although some find it a “slippery construct” because it is difficult to operationalize and always context-dependent (Jones, 2005; McGehee et al., 2010; Rodriguez-Giron and Vanneste, 2019). Social capital, as contemporarily conceptualized, was first defined by Bourdieu (1986) as “the aggregate of the actual or potential resources which are linked to possession of a durable network of more or less institutionalized relationships of mutual acquaintance and recognition – or in other words, to membership in group” (p. 286).

Social capital is conceptualized chiefly as a positive asset that leads to desirable outcomes in individuals or communities (Portes, 1998; Lin, 2002). For example, Jones (2005) found that a high level of social capital, manifested particularly in locals’ commitment to collective action, was instrumental to creating a successful community-run ecotourism camp in the Gambia. Similarly, Diedrich et al. (2019) reported that fishermen in Papua New Guinea with higher social capital perceived fewer entry barriers to start and operate sportfishing tourism microenterprises. Likewise, informal tourism microentrepreneurs in Thailand considered their social networks a vital source of business opportunities (Çakmak et al., 2019).

However, the “more is better” approach may be over-simplistic, considering that excessive embeddedness in a social network may be counterproductive in close-knit groups who may turn their backs to the outside (Portes, 1998; Agnitsch et al., 2006). Moreover, Uzzi (1996) posited that embeddedness yields positive returns only up to a certain point. Similarly, findings from a study with a small group of wildlife tourism microentrepreneurs suggest that a higher number of business ties diminishes the levels of trust, reciprocity, and togetherness toward other microentrepreneurs (KC et al., 2019b).

It is important to distinguish between two different forms of social capital, bonding, and bridging (Putnam, 1995). Bonding social capital is inherently “inward-looking,” and it promotes exclusive identities and homogeneous group characteristics, enabling access primarily to homogeneous resources common to every member. In turn, bridging social capital is inherently “outward-looking” and fosters connection to other people or groups who are different from each other in some way, therefore possessing heterogeneous resources from one another (Woolcock and Narayan, 2000).

Although KC et al. (2019a) found that business ties (i.e., bridging ties) were more prevalent than family and friends’ ties (i.e., bonding ties) among wildlife tourism microentrepreneurs, the latter were perceived as more important. Conversely, Rastrollo-Horrillo and Rivero Díaz (2019) reported that the ability to tap into resources provided by external agents was the most decisive factor on innovative behavior and success among tourism small- and microenterprises. Similarly, Martínez-Pérez et al. (2019) found that Spanish tourism enterprises with more bridging ties outside their business clusters performed significantly better on radical innovation. Finally, Li and Barbieri (2020) reported that networking opportunities afforded by agritourism associations are an important source of social capital and a privileged vehicle for exchanging information and resources among farmers. We argue that by extending farmers’ networks to different levels of social ties within the tourism business ecosystem, farmers will be better prepared to pursue agritourism opportunities.

### Permatourism

Rodriguez-Giron and Vanneste (2019) contend that social capital at destinations resides in three levels of social ties: internal ties in a group, bridging horizontal ties with new actors or groups, and linking vertical ties with actors or groups in power or control of critical resources. Similarly, Ferreira et al. (2021) posit that the success of grassroots tourism initiatives depends on the ability of microentrepreneurs to establish symbiotic relationships with actors in three tiers of the permatourism model, namely, other microentrepreneurs, formal private tourism sector actors, and support organizations (e.g., DMOs). Permatourism is a destination management framework that pursues the complementarity between formal private and public actors, local microentrepreneurs, and grassroots community social structures (Ferreira and Brothers, 2022). The model comprises five conceptual zones, 0 through 4, expanding outwards from the destinations’ pull factors, followed by local government branches and support agencies, the tourism formal sector, the informal microentrepreneurial sector, and, finally, residents.

On the importance of the symbiosis with the formal sector, Karampela et al. (2019) reported that agritourism operators in two remote Greek islands rely on the influx of “conventional” tourists due to limited accessibility and high travel costs to and from those locations. In North Carolina, a university-led project energized county-level networks of agritourism microentrepreneurs through developing and nurturing relationships with actors in the Zone 1 (e.g., DMO, Cooperative Extension, University), Zone 2 (e.g., chefs, restaurateurs, and hoteliers), and Zone 3 (e.g., established and aspiring agritourism microentrepreneurs; Morais et al., 2017a).

However, the literature also points out some potential limitations of farmers’ network ties. In Zone 1, destination managers are often unable to articulate the merits of agritourism to tourists and fail to incorporate such activities in the mainstream tourism product (McGehee, 2007; Barbieri et al., 2019). Likewise, small business development and agriculture extension officers, unfamiliar with the activity, may be ill-equipped to support farmers wanting to diversify into farm-based tourism (Arroyo et al., 2013; Ferreira et al., 2020). In Zone 2, while collaborations between chefs and farmers are becoming increasingly popular, such events are often organized, managed, and promoted by chefs, relegating the farmer to a secondary and passive role (Zaneti, 2017). Also, in a study conducted by Curtis et al. (2008), evaluative assessments conducted with farmers and local chefs revealed that both groups lacked appropriate information concerning production possibilities, market needs, and customer preferences. In Zone 3, research suggests that, on the one hand, a high number of network ties are associated with lower levels of trust among tourism microentrepreneurs (KC et al., 2019b). On the other, the informality of the networks tends to favor the importance of family ties in lieu of business ties (KC et al., 2019a).

In sum, research suggests that networks cutting across different tiers of the tourism business ecosystem gives farmers access to heterogeneous resources and information crucial to the success of their tourism operations. Moreover, we argue that these business ties are an equally important source of entrepreneurial self-efficacy by creating opportunities to model entrepreneurial behavior from successful individuals.

### Self-Efficacy

Self-efficacy, defined as one’s belief in one’s ability to succeed in a target behavior, is a dominant theoretical paradigm used to explain people’s motivation, effort, and perseverance in a task (Bandura, 1977). Self-efficacy theory holds that if people perceive themselves to be capable of accomplishing certain activities, they are more likely to undertake them in the future (Alkire, 2005). Moreover, self-efficacy will also influence an individual’s level of motivation, as reflected in how much effort one will exert in a task and how long one will persevere in the face of obstacles (Bandura, 1980). Later work has conceptualized self-efficacy in terms of its internal and external dimensions (Etter et al., 2000; De Geus et al., 2008; Thatcher et al., 2008; Martinez et al., 2010), the latter allowing for an emphasis on situations wherein an individual is able to persevere even in the face of suboptimal external stimuli. For example, in a study on work commuting in Flanders, De Geus et al. (2008) reported that individuals with higher external self-efficacy scores were more likely to bike to work even under adverse weather conditions or on days where they had to run more errands.

Boyd and Vozikis (1994) pioneered the application of self-efficacy in the context of entrepreneurship. Entrepreneurial self-efficacy (ESE) is defined as one’s belief in one’s ability to perform entrepreneurship-related tasks, and findings indicate it is a multidimensional construct (Chen et al., 1998; De Noble et al., 1999; McGee et al., 2009; Moberg, 2013). Ferreira et al. (2018) adapted the construct to tourism microentrepreneurship defining it as one’s belief in one’s ability to successfully perform the various roles and tasks of microentrepreneurship in the tourism e-business sector. In addition, confirmatory factor analysis on a sample of 300 urban tourism microentrepreneurs suggested that tourism e-microentrepreneurial self-efficacy (TeMSE) encompasses five dimensions, namely, *Pursuing Innovation*, *Marshaling Resources*, *Adapting to Externalities*, *Aligning Core Purpose with Self*, and *e-Marketing* (Ferreira et al., 2018).

Examining entrepreneurial self-efficacy among farm tourism microentrepreneurs is central to this study. Firstly, because of the amply documented positive relationship between self-efficacy and entrepreneurial intention (De Noble et al., 1999; Markman et al., 2002, 2005; Arenius and Minniti, 2005; Wilson et al., 2007; Wu and Tian, 2022) and secondly because of the mediating role of self-efficacy between social capital and successful behavior (Liñán and Santos, 2007; Brouwer et al., 2016).

**Figure 1:**
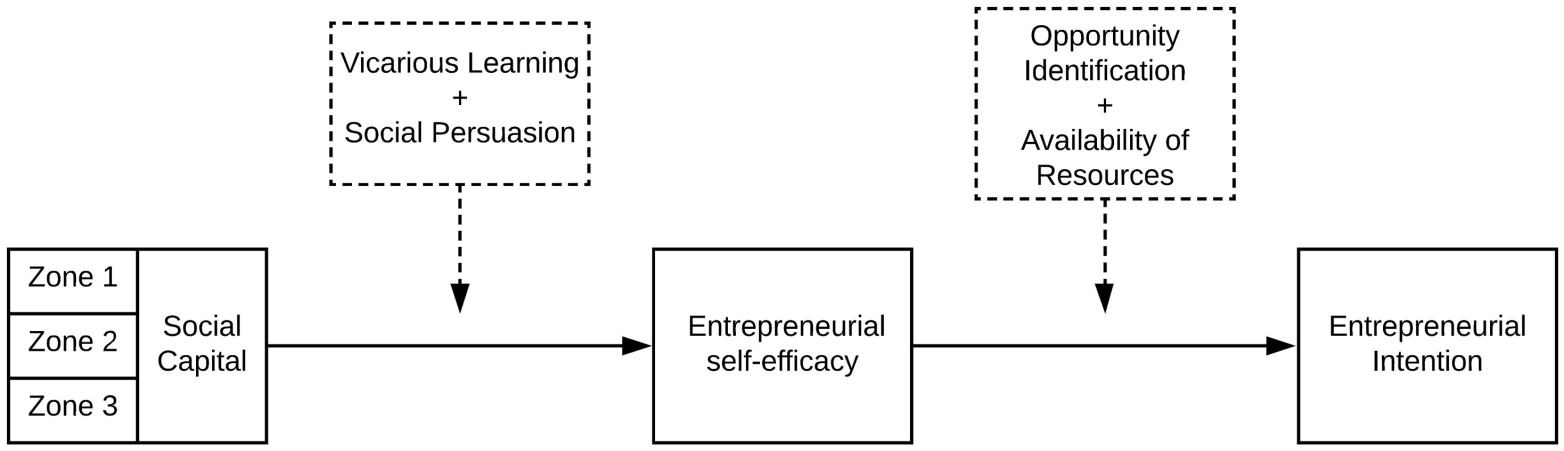
Antecedents of entrepreneurial intention.

Figure 1 shows the self-efficacy generating mechanisms in the studied context. There are four sources of self-efficacy: enactive mastery experiences, modeling or vicarious learning, social persuasion, and physiological factors (Bandura, 1982). We argue that extended network ties with actors across different tiers of the tourism business ecosystem provide opportunities for (1) vicarious learning/modeling and (2) social persuasion (e.g., encouragement and reassurance), which are theorized to influence farmers’ TeMSE. Although the actual effect of social persuasion is found to be short-lived, we argue that select actors in the tourism business ecosystem (e.g., famous chefs; proficient restauranteurs; and hotel managers) can be “credible persuaders” (Bandura, 2008, p. 169), by virtue of their prominence in society and perceived successful business endeavors.

### Hypotheses

Past studies show a positive association between social capital and entrepreneurial self-efficacy and between the latter and entrepreneurial intention. Hence, we argue that network ties across the permatourism system can elevate farmers’ TeMSE levels and, consequently, their intentions to start or add value to an existing farm tourism microenterprise. Hence, the research hypotheses were developed as follows:

*H1*

*H1a*: There is a positive relationship between bridging social capital and tourism microentrepreneurial internal self-efficacy.

*H1b*: There is a positive relationship between bridging social capital and tourism microentrepreneurial external self-efficacy.

*H2*

*H2a*: There is a positive relationship between tourism microentrepreneurial internal self-efficacy and tourism microentrepreneurial intention.

*H2a*: There is a positive relationship between tourism microentrepreneurial external self-efficacy and tourism microentrepreneurial intention.

## Materials and Methods

### Participatory-Action Research

This study subscribes to a transformative worldview (Nelson and Prilleltensky, 2010), wherein the research team and study participants are equals in the research process and pursue a shared horizon in which tourism is an enabler of socioeconomic prosperity in the community. Accordingly, literature in community psychology calls for transformational scholarship in the social sciences, stressing the limitations of conventional mainstream psychology and other social sciences (Wisner et al., 1991; Seymour-Rolls and Hughes, 2000; Davis, 2008). Hence, participatory-action research (PAR) goes beyond the boundaries of traditional paradigms of research that call for the least disturbance in the study environment, being instead a method primarily concerned with bringing about social change to participants in the research process (Seymour-Rolls and Hughes, 2000; Nelson and Prilleltensky, 2010). Importantly, PAR can use quantitative and/or qualitative methods.

**Figure 2:**
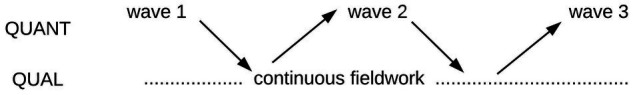
Multi-wave survey design.

This study was part of a larger longitudinal research project wherein quantitative and qualitative data are linked by way of a multi-wave survey design (Figure 2), conducted in parallel with continuous fieldwork (Miles and Huberman, 1994; Flick, 2014). Under this mixed-methods approach, (1) qualitative approaches (e.g., formal interviews, impromptu conversations, and community meetings) were used to co-generate research questions, develop the questionnaire, and refine scales; (2) quantitative methods were utilized to test the hypotheses empirically; and (3) qualitative data (e.g., interviews transcripts, field notes, and reflexive memos) were used to triangulate the data from the survey.

### Qualitative Data

All research team members have extensive experience working and conducting research with rural communities in North Carolina. In 5 years, the lead researcher made over 50 individual visits to farms, interviewed over 20 chefs involved in the local foods movement, met with upwards of 30 Cooperative Extension directors and destination managers, in the scope of multiple participatory-action research projects. In addition, the two lead researchers kept a reflexive journal throughout the research process where they recorded thoughts, highlights from impromptu conversations, and field observations (for examples, see Flick, 2014; Nazariadli et al., 2019).

### Quantitative Data

This study aims to better understand predictors of farmers’ intention to pursue direct sales of product and experiences to visitors; therefore, the research team developed a database of North Carolina farms with varying degrees of involvement in the direct sales of products and experiences to the public. The database was curated by Cooperative Extension staff members, listing 1,200 farms publicly listed in web databases like Appalachian Grown, visit NC farms, and Carolina Farm Stewardship, and featured in the web pages of select farm-to-table restaurants.

Following IRB approval, the online survey was sent to select partners in rural North Carolina for pilot-testing. After revisions were made to the instrument, we sent out an email inviting farmers to participate in the survey. Subsequently, we scheduled four reminders to fall on different days of the week, including weekends, and on different times to foster a better response rate. At the end of the survey, participants were invited to enter their email addresses to qualify for a prompt participation incentive of a $25 gift certificate to a farm supply store. Specifically, the first 100 participants to complete the survey and volunteer their email addresses were notified of their gift certificate *via* email, and the gift certificates sent to them *via* snail mail promptly after culminating data collection.

**Figure 3:**
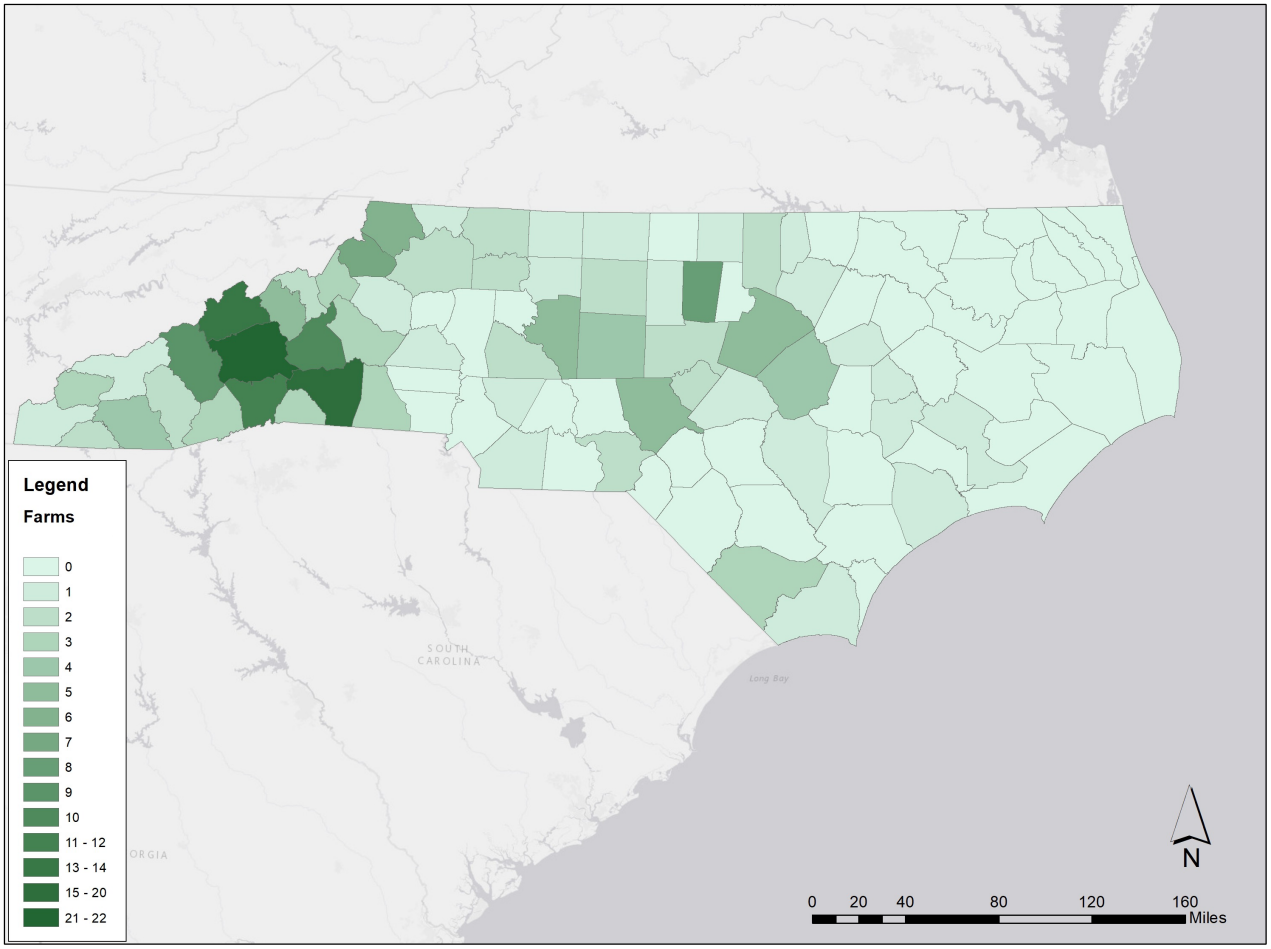
Number of study participants per county.

The web-based administration of the survey yielded 273 responses, corresponding to a 23% response rate, in line with expected response rates of survey research in a region like North Carolina (Groves et al., 2011). Cases with missing data or evidence of careless response (Meade and Craig, 2012) were deleted, bringing the final count to 207 valid responses. Response rates across the many counties in North Carolina were relatively homogeneous; however, the sampling frame included more participants from the Western counties, so, as illustrated in Figure 3, farmers from western counties were more represented in the study sample.

### Instruments and Data Analysis

The proposed model includes three constructs. Individually owned bridging social capital was adapted from Chen et al. (2008) to the context of farm tourism microentrepreneurship to capture farmers’ ties with actors in the three zones proposed by the permatourism model. Hence, we developed three subscales, for Zones 1–3, respectively, with 5, 4, and 4 items, using a five-point Likert-like scale ranging from 1 = “none” to 5 = “all.” Tourism microentrepreneurial self-efficacy was adopted directly from Ferreira et al. (2018). The TeMSE scale is originally a set of five subscales, representing five facets of the construct. However, because it was developed with a sample of urban microentrepreneurs, it was expected that the scale’s underlying structure might change in the context of farm tourism microentrepreneurship. Hence, we conducted exploratory factor analysis before validating the model, as suggested by Garson (2015). Consequently, principal component analysis with varimax rotation on the 13 items revealed a two-factor underlying structure (Garson, 2013). The first factor included eight items relating to perceived internal entrepreneurial capabilities (e.g., innovation, marketing, and leadership), whereas the second five-item factor reflected capabilities related to coping with external stimuli (e.g., labor shortages and understanding legislation). These two subscales use a five-point Likert-like scale ranging from 1 = Strongly Disagree to 5 = Strongly Agree. Finally, entrepreneurial intention was adapted from Chen et al. (1998), following DeVellis's (1991) recommendations, culminating in an eight-item Likert-like scale ranging from 1 = Extremely Unlikely to 5 = Extremely Likely (see Table 1 for a list of all items).

**Table 1:** Farmers demographic and agritourism profile.

Demographic characteristics	*n*	Percent
*Gender* (*n* = 206) *q7.3*		
Male	85	41.1
Female	117	56.5
Other	1	0.5
Prefer not to respond	3	1.4
*Ethnicity* (*n* = 206)		
Native American	3	1.4
Caucasian/White	193	93.2
Hispanic/Latino	1	0.5
Other	4	1.9
Prefer not to respond	5	2.4
*Age* (*n* = 206)		
Mean (in years)		(53.6)
*Education level*		
High school graduate (high school diploma or equivalent including GED)	11	5.3
Some college but no degree	31	15.0
Associate degree in college (2-year)	25	12.1
Bachelor’s degree in college (4-year)	87	42.0
Master’s degree	45	21.7
Doctoral degree	4	1.9
Professional degree (JD, MD)	3	1.4
*On and Off-farm employment* (*n* = 207)		
Full-time employee off the farm	44	21.3
Part-time employee off the farm	21	10.1
Run another business	32	15.5
Homemaker (care for household)	23	11.1
Student	1	0.5
Retired from previous career	43	20.8
Other	7	3.4
Full-time farmer—no other employment	79	38.2
*Position in the farm* (*n* = 207)		
Owner/Co-owner	192	92.8
Farm manager	8	3.9
Full-time farm worker	1	0.5
Seasonal contract worker	1	0.5
Other	5	2.4
Agritourism profile		
*Farm* (*n* = 207)		
Size of farm (in acres)		(87.9)
Size of farmed land (in acres)		(48)
*Importance on financial viability* (*n* = 207)		
Sales of product at the farm (farm stand, U-pick)		
Not important at all	48	23.2
Of little importance	26	12.6
Of average importance	30	14.5
Very important	43	20.8
Absolutely essential	60	29.0
Sales of experiences/tours to farm visitors (workshops, farm dinners, farm stays, etc.)		
Not important at all	49	23.7
Of little importance	29	14.0
Of average importance	35	16.9
Very important	39	18.8
Absolutely essential	55	26.6
*Percentage of total farm revenue* (*n* = 207)		
Sales of product directly to chefs and restaurants	-	13.9
Sales of product directly to visitors of your farm, farm stand or farmers market	-	41.2
Sales of experiences/tours/stays to visitors of your farm	-	14.4

To test the validity of the measures and the structural relationships between constructs (Garson, 2015; Meyers et al., 2017), we conducted confirmatory factor analysis (CFA) and structural equation modeling (SEM) using AMOS within the statistical package for the social sciences (SPSS). Regarding sample size, recommendations vary in the literature, based on the number of parameters to be estimated, the number of variables in the model, and missing data. This study follows the recommendation by Garson (2015) of a sample of at least 200 to provide significance tests with adequate power.

Several criteria were used to assess the goodness-of-fit to the observed data. The first was the chi-square statistic; however, due to its sensitivity to sample size, the following criteria recommended by Garson (2015) were also used as: (1) a ratio of chi-square to degrees of freedom [CMIN/DF] less than 2.0 (Byrne, 2001); (2) two incremental indices, Comparative Fit Index (CFI) and Tucker–Lewis Index (TLI) greater than or equal to 0.90 (Hu and Bentler, 1999); (3) a Standardized Root Mean Square Residual smaller than or equal to 0.07 (Hu and Bentler, 1999); and Root Mean Square of Approximation (RMSEA) smaller than or equal to 0.07 (Browne and Cudeck, 1993). The CFI and TLI test the model against a null or independence model, which assumes there are no covariances among the observed values in the population. The SRMR compares the actual sample correlation matrix to the population correlation matrix resulting from the model and represents the average of the standardized residuals between the two (Brown, 2015). The RMSEA evaluates a hypothesized model by comparing it to a model with perfect fit and takes into account sample size and model complexity.

### Reliability, Convergent Validity, and Discriminant Validity

CFA also provides a stringent test for construct validity. Accordingly, composite reliability (CR) was used to measure the internal consistency of the factors, where values greater than 0.70 indicate good reliability (Hair et al., 2010), although values greater than 0.6 may be acceptable for exploratory research (Garson, 2016). Discriminant validity is achieved if average variance extracted (AVE) is greater than the maximum shared squared variance (MSV) or the average shared squared variance (ASV); for achieving convergent validity, AVE should be equal or greater than 0.50 and lower than CR (Hair et al., 2010).

## Results

### Respondents’ Socio-Demographic Profile and Their Engagement in Agritourism

According to Table 1, participating farmers mainly were female (56.5%), white (93.2%), and in their mid-adulthood (53.6 years old), which is consistent with demographic trends among farm tourism microentrepreneurs in North Carolina (Xu et al., 2014). More than two-thirds (38.2%) of the participants worked exclusively on-farm, and most (42%) held a bachelor’s degree. Importantly, for the goal of this study, the vast majority (92.8%) indicated being either the owners or co-owners of the farm.

Regarding their engagement in farm tourism, most farmers indicated that sales of product at the farm (29%) and sales of experiences to farm visitors (26.6%) were absolutely essential to the financial viability of the farm. However, the distribution of responses appears to be bimodal, with approximately the same number of farmers indicating these activities are not important at all. Also, despite apparently being important to farmers, revenue from sales of farm experiences was on average only 14.4% of total farm revenue.

### Measurement Model

CFA revealed that the measurement model had an acceptable fit with the data: *χ*^2^ (265) = 412.9, CMIN/DF = 1.556, CFI = 0.950, TLI = 0.944, SRMR = 0.075, RMSEA = 0.052.

Table 2 shows that internal self-efficacy, external self-efficacy, and intention had adequate reliability, convergent validity, and discriminant validity. However, second-order variable social capitals’ convergent validity and reliability values fell short of the cutoff values proposed by Hair et al. (2010). This could be because the first-order variables, Zones 1–3, represented different tiers of social capital, thus unlikely to be highly correlated (Garson, 2015). Hence, according to the same author, “if SEM analysis shows good fit, this is an indication that indicator variables reflect the latent variables they are supposed to and that the latent variables are different from each other. That is, CFA establishes convergent and divergent validity in the proposed model” (Garson, 2015, p. 24). In the face of these mixed results, we further assessed the internal consistency construct validity of each first-order factor through the Cronbach-alpha statistic and concluded that they are acceptable for exploratory purposes.

**Table 2:** CFA of complete measurement model.

	Scale and item description	Mean	***R***	Error	AVE	CR	Alpha
	**Individually owned social capital**				0.345	0.509	
	Permatourism zone 1						0.87
	*Of all the governmental, professional, and civic organizations in your community (*e.g.*, tourism development authority, chamber of commerce, cooperative extension, farmers associations)...*						
Q4.2.3	… how many represent your rights and interests?	2.65	0.770^*^	0.349			
Q4.2.4	… how many of them seem committed to your success?	2.76	0.906^*^	0.204			
Q4.2.5	… how many do you think would help you upon your request?	3.00	0.831^*^	0.367			
	Permatourism Zone 2						0.57
	*Of the many chefs, restaurateurs, and hoteliers in your community...*						
Q4.3.2	… how many of them buy farm products from you?	1.91	0.640^*^	0.349			
Q4.3.3	… how many of them seem committed to your success as a farmer and business partner?	2.24	0.956^*^	0.097			
Q4.3.4	… how many of them do you think would help you upon your request?	2.35	0.822^*^	0.334			
	Permatourism Zone 3						0.78
	*Of all the farmers in your community...*						
Q4.4.2	… how many of them collaborate with you?	2.40	0.600^*^	0.463			
Q4.4.3	... how many of them seem to share your views on selling product and experiences to visitors at the farm?	2.64	0.787^*^	0.345			
Q4.4.4	...how many of them do you think would help you upon your request?	3.10	0.815^*^	0.371			
	**Internal self-efficacy**				0.616	0.918	
Q3.2.1	I am able to form partnerships with other businesses to strengthen my own tourism business.	3.71	0.742^*^	0.400			
Q3.2.2	I am able to use internet to market my tourism business.	3.97	0.738^*^	0.431			
Q3.2.3	I am able to discover ways to improve the appeal of tourism experiences I offer.	3.68	0.836^*^	0.258			
Q3.2.5	I am able to create experiences that fulfill tourists’ interests.	3.75	0.894^*^	0.179			
Q3.2.6	I am able to be myself while providing good customer service to tourists.	4.14	0.743^*^	0.361			
Q3.3.6	I am able to get others to believe in my plans for my tourism business.	3.51	0.675^*^	0.409			
Q3.2.7	I am able to use internet to engage customers and business peers with my tourism business.	3.86	0.843^*^	0.258			
	**External self-efficacy**				0.512	0.806	
Q3.3.1	I am able to understand tourism legislation that applies to my tourism business.	3.25	0.658^*^	0.511			
Q3.3.2	I am able to get the type of insurance I need for my tourism business.	3.40	0.775^*^	0.374			
Q3.3.3	I am able to understand what my liability is in case of an accident involving tourists.	3.52	0.783^*^	0.354			
Q3.2.4	I am able to find helpers for my tourism business when I need to tackle a problem or opportunity.	3.17	0.633^*^	0.645			
	**Intention**				0.652	0.903	
	*In the next 12 months, how likely are you to...*						
Q6.2.2	… start or expand sales of farm experiences to farm visitors (e.g., tours, workshops, farmstays)?	3.33	0.783^*^	0.729			
Q6.2.5	… organize events at your farm (e.g., weddings, farm dinners, hayrides, corn mazes)?	2.97	0.735^*^	1.106			
Q6.2.7	… explore more avenues to diversify your farm’s revenue by attracting visitors?	3.51	0.869^*^	0.400			
Q6.2.8	… seek ways to make your farm an integral part of the tourism offerings of your community?	3.39	0.937^*^	0.211			
Q6.2.9	… participate as a host in a regional farm tour event (e.g., cycle to farm, art and farm)?	3.29	0.689^*^	0.878			

**N* = 207; Measure of model fit: chi-square (265) = 412.9, CMIN/DF = 1.556, CFI = 0.950, TLI = 0.944, SRMR = 0.075, RMSEA = 0.052; R, standardized regression coefficient; AVE, average variance extracted; CR, construct reliability; and *α*, Cronbach’s alpha*.

* *p = 0.001*.

### Structural Model and Test of Hypotheses

**Figure 4:**
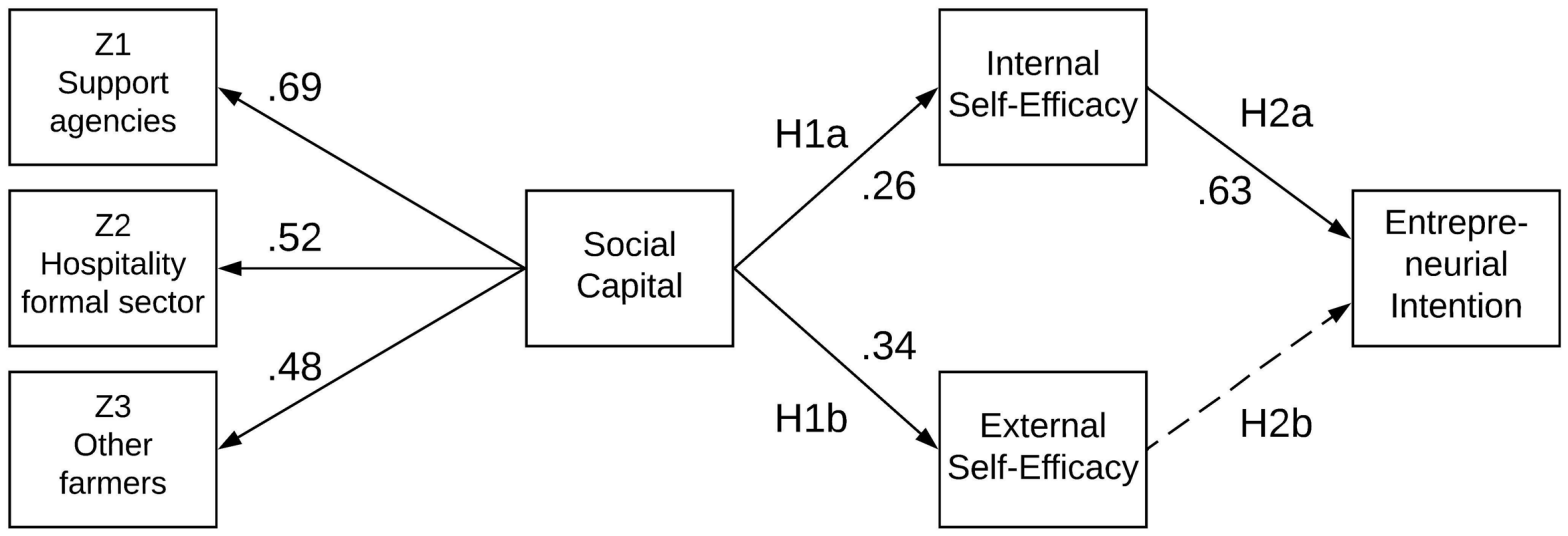
Structural equation model.

Following the validation of the measurement model, we used the magnitude and the significance of the relationships between latent variables (Figure 4) to test the hypotheses. First, the structural model’s goodness-of-fit was assessed using the same statistics utilized for the CFA. The SEM revealed adequate model fit: χ^2^ (267) = 470.4, CMIN/DF = 1.762, CFI = 0.931, TLI = 0.923, SRMR = 0.1253, RMSEA = 0.061. Although the structural model showed a lower fit than the measurement model, this was somewhat expected as recursive models cannot improve fit compared to the CFA (Hair et al., 2010).

Hypothesis H1a and H1b tested the relationship between social capital and self-efficacy. Given that social capital had a positive and significant relationship both with internal self-efficacy (*β* = 0.26; *p* < 0.05) and external self-efficacy (*β* = 0.34; p < 0.05), both hypotheses H1a and H1b were supported by the study (Table 3). This finding supports previous reports in the educational context where social capital was also positively associated with self-efficacy (Liñán and Santos, 2007; Brouwer et al., 2016).

**Table 3:** Testing of hypotheses.

Hypotheses	Hypothesized relationship	St. regression weights	***P***	Support for hypothesis
H1a	Social capital ➔ internal self-efficacy	0.26^*^	0.017	Y
H1b	Social capital ➔ external self-efficacy	0.34^*^	0.007	Y
H2a	Internal self-efficacy ➔ entrepreneurial intention	0.63^*^	0.000	Y
H2b	External self-efficacy ➔ entrepreneurial intention	0.01	0.883	N

**N* = 207; Measure of model fit: *χ*^2^ (267) = 470.4, CMIN/DF = 1.762, CFI = 0.931, TLI = 0.923, SRMR = 0.1253, RMSEA = 0.061*.

* *p = 0.05*.

In addition, this finding is also supported by qualitative data. For example, research team members took part in several meetings led by an agritourism specialist from the NC Department of Agriculture (Zone 1) in the scope of the counties’ participation in an app promoting agritourism experiences. The specialist addressed intricacies of farm tourism, including aspects related to self-representation, programing, and legal matters, which stimulated ideas for innovation and clarity on how farmers can seek help to cope with externalities (i.e., internal and external entrepreneurial self-efficacy, respectively). Additionally, during a field visit with farmer and tourism microentrepreneur, he reiterated the importance of developing partnerships with formal tourism industry partners (Zone 2), indicating that “*[the chef] wanted to get to know me right off the bat… that was important because, especially starting out, we do not have relationships with anybody… we kinda do not know what we are doing… and we look to guidance a lot from the people who have been in the industry for a long time*.” Finally, the importance of bridging ties between peer microentrepreneurs (Zone 3) was evident in several counties in which we work, when a farmer who was the pioneer of agritourism in the county decades ago is revered by fellow farmers as a source of inspiration and knowledge and was instrumental in kick-starting a network of farm tourism microentrepreneurs.

Hypothesis H2a and H2b tested the relationship between self-efficacy and entrepreneurial intention. Internal self-efficacy had a positive and significant relationship with intention (*β* = 0.63; *p* < 0.05); therefore, hypothesis H2a is supported by the data (Table 3). This finding also supports previous research in entrepreneurship where self-efficacy was also positively associated with entrepreneurial intention (De Noble et al., 1999; Markman et al., 2002, 2005; Arenius and Minniti, 2005; Wilson et al., 2007). However, unexpectedly, external self-efficacy was not significantly associated with entrepreneurial intention. This counter-intuitive finding is partially supported by qualitative data, which suggests that most farmers do engage in agritourism regardless of their knowledge of the regulatory framework governing it. Accordingly, farm tourism microentrepreneurs have to cope with ambiguity and uncertainty due to unclear policy and regulations. For example, one farmer complained that he had been denied a license to turn his log cabin into a Bed & Breakfast operation, although he could rent it out on Airbnb. Another farmer grieved that “*It was a nightmare to have them approve my project. Every time they came to inspect, somehow they always managed to find something wrong*.” The inability to find competent farm helpers was also mentioned during our fieldwork as a challenge to farm tourism microentrepreneurship. Yet, it is primarily an indirect impact, in the sense that owners cannot engage in agritourism in cases where they cannot delegate agricultural production-related tasks that drain all their time. One particularly active farmer in the hosting of weddings and farm dinners complained that she had gone through four immigration attorneys and spent over $20,000 trying to obtain a green card for her foreign farm manager, which would make her free to pursue other entrepreneurial opportunities.

## Discussion

Over the last few decades, small farmers in North Carolina (as in other US regions) have struggled to adapt to progressive challenges to their business conditions (McKee, 2018). Some have embraced emerging opportunities to sell products directly to consumers with varying degrees of success, and some have explored new revenue streams by offering product and experiences to visitors (Barbieri and Mahoney, 2009; Ferreira et al., 2020). The conceptual framework described earlier explains that entrepreneurs’ involvement with and perseverance in new business activity are highly dependent on their self-efficacy, and their self-efficacy is highly dependent on the role modeling and affirmation they receive from reference groups. Accordingly, the purpose of this study was to examine the extent to which farmers’ intention to be involved in tourism microentrepreneurial activities is influenced by their ties to other business partners and public agencies.

We posited that low bridging social capital limits opportunities for farmers to model entrepreneurial behavior from business partners and to receive positive encouragement from supporting organizations, thus impeding the development of the farmers’ microentrepreneurial self-efficacy and their intentions to start tourism enterprises on the farm. We drew on social cognitive theory (Bandura, 1977), social capital theory (Bourdieu, 1986), and permatourism (Ferreira et al., 2021) to propose that resources should be directed to programs and initiatives that foment and nurture symbiotic relationships between farmers, business partners, and public agencies, to boost farmers’ social exchange opportunities across power and authority gradients in the tourism business ecosystem (Li and Barbieri, 2020). Such exchanges are important sources of self-efficacy through several mechanisms, which positively influence the development of entrepreneurial intentions.

Consistent with previous literature, social capital was positively associated with self-efficacy in this study. To our knowledge, however, this was the first time this relationship was empirically examined in the context of agritourism entrepreneurism. It suggests that informal networks, more welcoming and less threatening than rigid and bureaucratic formal structures, can be equally effective in nurturing and supporting farm tourism microentrepreneurs (Karampela et al., 2019). Hence, destination management organizations may need to loosen their formal requirements for partnerships and collaborations with informal players in the interest of destinations’ uniqueness and competitiveness. One such constraint we have observed in the field is the requirement of DMOs to support only businesses that overtly contribute to overnight stays (the source of occupancy tax; Freeze, 2021). Such a shift in institutional approach would help DMOs better serve the community and not just the formal lodging industry (Destinations International, 2019; Buhalis, 2022).

In addition to being positively associated with social capital, internal self-efficacy was a positive and significant predictor of microentrepreneurial intention. This suggests that self-efficacy is a central tenet in the permatourism model, as it seems to be the mechanism through which tourism microentrepreneurs convert the resources embedded in their networks into intention to pursue tourism business. In this regard, Bandura (2006) posits that “the value of psychological theory is judged not only by its explanatory and predictive power, but by its operational power to make change” (p. 319).

In turn, external self-efficacy was not significantly associated with intention. In a rare study where this link was also non-significant, Hill et al. (1996) argue that managers may have a small degree of control over particular tasks in their purview, and therefore, efficacy perceptions are irrelevant to their behavioral intention in such cases. It is important to note that, while they used a unidimensional four-item self-efficacy scale, at least two of the items appear to capture the external dimension of self-efficacy (i.e., “The number of events which could prevent me from (…) are: *very few* to *very numerous*” and “How much control have you over factors that are involved in (…)? *no control* to *complete control*”). Hence, the specificity of the entrepreneurial tasks encapsulated in the items of either TeMSE subscales may explain why one dimension was significantly associated with entrepreneurial intention and the other was not.

Nevertheless, the SEM path between social capital and external self-efficacy was higher than with internal self-efficacy. It would thus be reasonable to expect the path between external self-efficacy, farmers’ perceived ability to comply with industry regulations, and entrepreneurial intention to be also statistically significant. To this point, our experience in the field suggests that farmers, out of choice or need, do not necessarily wait until they have a perfect domain of farm tourism’s legal landscape to start their ventures. In this vein, Bosworth and Farrell (2011) note that a fair share of rural entrepreneurship is driven by necessity, rather than ambition or skill, by financially constrained individuals (i.e., non-entrepreneurs). For many, in the face of rising property tax or outstanding bank loans, monetizing the family’s real estate through tourism is the only way viable alternative to bankruptcy or a sell-off.

Furthermore, compared to internal self-efficacy, the observed lower values of external self-efficacy suggest that information is ambiguous in respect to licenses, insurance, and taxes due for a tourism business. While farmers’ concern about liability for personal injuries of participants, as well as their revindication for clear instructions from agritourism regulatory bodies, is not new (Centner, 2010; Leff, 2011), this study shows that it is not an impeding factor for new venture creation. Hence, agencies tasked with providing agritourism training focusing on insurance, liability, or risk management (Infante-Casella et al., 2018) should target both nascent and established farm tourism microentrepreneurs.

### Limitations

Although widely used in tourism research, many have found social capital challenging to operationalize because it may mean different things in different contexts (Jones, 2005; McGehee et al., 2010; Rodriguez-Giron and Vanneste, 2019). In this vein, the adapted the scale by Chen et al. (2008) to the context of this study may need further refinement in the face of the mixed reliability and convergent validity analyses results. Also, our findings may not be generalizable to other countries or even other states in the United States. For example, Chiodo et al. (2019) found singular differences and national specificities in agritourism case studies in the United States, Brazil, Italy, and France. Moreover, Karampela et al. (2019) reported that geographic characteristics in insular or continental Greece determined the type of agritourism units, the scale of the operation, and the strength of the networks. Finally, although our sample is larger than the lower bound of 200 cases defined by Garson (2015), follow-up studies should consider larger samples for improved statistical robustness.

### Further Research

While this study suggests that partnerships with formal industry partners, like chefs and restaurateurs, may constitute important sources of social capital and self-efficacy among farmers, we need to recognize that the exchange of non-economic assets is not unidirectional (Zaneti, 2017; LaPan et al., 2021). Thus, future research should look into how chefs and restaurateurs capitalize on partnerships with local farmers to improve their businesses. Namely, the enhanced level of transparency (Bhaduri and Ha-Brookshire, 2011) that farm visits bring to the restaurant’s practices and the alignment with important core values that resonate with the patronage should be examined.

Some processes and mechanisms of farm tourism microentrepreneurship appear to fall under what Baker and Nelson (2005) and Fisher (2012) describe as entrepreneurial bricolage. Entrepreneurs employ this strategy to overcome resource-constrained environments by recycling and repurposing available material and human resources to create something from nothing. In particular, one area where farm tourism microentrepreneurs appear to enact bricolage is in the institutional and regulatory environment, “by refusing to enact limitations with regard to many ‘standards’ and regulations, and by actively trying things in a variety of areas in which entrepreneurs either do not know the rules or do not see them as constraining, bricolage creates space to ‘get away with’ solutions that would otherwise seem impermissible” (Baker and Nelson, 2005, p. 349). Hence, we believe that entrepreneurial bricolage is a promising conceptual framework for future studies.

## Conclusion

This study used a mixed-methods approach to explore the extent to which farm-based tourism entrepreneurial intentions can be predicted by the strength of bridging ties with public sector support agencies (e.g., extension agents and DMOs), formal private sector partners (e.g., farm-to-table chefs), and with other peers (e.g., other farmers involved in farm tourism microentrepreneurship). Importantly, these three levels of potential farm partnerships represent, respectively, Zones 1–3 under the permatourism paradigm (Ferreira et al., 2021). In the model proposed, social capital predicts both dimensions of entrepreneurial self-efficacy, internal and external factors. However, only internal self-efficacy has a significant relationship with entrepreneurial intention, which suggests that farmers’ intention to engage in farm tourism microentrepreneurship is independent of their perceived efficacy in understanding the industry’s regulation or getting adequate liability coverage. In addition, lower scores in this dimension also suggest that farmers may decide to go forward with their entrepreneurial projects despite apparently being unprepared to deal with essential aspects of the business. Not only is this uncertainty problematic because of potential legal implications of non-compliance with the regulatory frameworks, but also because it may lead to venture failure and decreased entrepreneurial self-efficacy (which may hold farmers back in the future). In totality, this study asserts that multilateral tourism initiatives involving actors across different permatourism zones can increase farmers’ intention to develop the supply of farm tourism experiences. At the same time, evidence equally suggests that formal training focusing on legal aspects of agritourism is necessary for the success of farm tourism, given that these competencies, due to their complexity and ambiguity, may be challenging to acquire on the job.

## Data Availability Statement

The raw data supporting the conclusions of this article will be made available by the authors, without undue reservation.

## Ethics Statement

The studies involving human participants were reviewed and approved by the North Carolina State University Institutional Review Board Office. The patients/participants provided their written informed consent to participate in this study.

## Author Contributions

All authors listed have made a substantial, direct, and intellectual contribution to the work and approved it for publication.

## Funding

This study was funded by the United States Department of Agriculture’s (USDA) Agricultural Marketing Service through grant AM170100XXXXG110. Article processing fees were supported by Arizona State University.

## Conflict of Interest

The authors declare that the research was conducted in the absence of any commercial or financial relationships that could be construed as a potential conflict of interest.

## Publisher’s Note

All claims expressed in this article are solely those of the authors and do not necessarily represent those of their affiliated organizations, or those of the publisher, the editors and the reviewers. Any product that may be evaluated in this article, or claim that may be made by its manufacturer, is not guaranteed or endorsed by the publisher.
